# Highly Strain‐Stable Intrinsically Stretchable Olfactory Sensors for Imperceptible Health Monitoring

**DOI:** 10.1002/advs.202302974

**Published:** 2023-08-23

**Authors:** Guodong Zhao, Jing Sun, Mingxin Zhang, Shanlei Guo, Xue Wang, Juntong Li, Yanhong Tong, Xiaoli Zhao, Qingxin Tang, Yichun Liu

**Affiliations:** ^1^ Center for Advanced Optoelectronic Functional Materials Research and Key Lab of UV‐Emitting Materials and Technology of Ministry of Education Northeast Normal University Changchun 130024 P. R. China

**Keywords:** gas sensor, intrinsically stretchable, strain‐insensitive

## Abstract

Intrinsically stretchable gas sensors possess outstanding advantages in seamless conformability and high‐comfort wearability for real‐time detection toward skin/respiration gases, making them promising candidates for health monitoring and non‐invasive disease diagnosis and therapy. However, the strain‐induced deformation of the sensitive semiconductor layers possibly causes the sensing signal drift, resulting in failure in achievement of the reliable gas detection. Herein, a surprising result that the stretchable organic polymers present a universal strain‐insensitive gas sensing property is shown. All the stretchable polymers with different degrees of crystallinity, including indacenodithiophene‐benzothiadiazole (PIDTBT), diketo‐pyrrolo‐pyrrole bithiophene thienothiophene (DPPT‐TT) and poly[4‐(4,4‐dihexadecyl‐4H‐cyclopenta[1,2‐b:5,4‐b′]dithiophen‐2‐yl)‐alt‐[1,2,5]thiad‐iazolo [3,4‐c] pyridine] (PCDTPT), show almost unchanged gas response signals in the different stretching states. This outstanding advantage enables the intrinsically stretchable devices to imperceptibly adhere on human skin and well conform to the versatile deformations such as bending, twisting, and stretching, with the highly strain‐stable gas sensing property. The intrinsically stretchable PIDTBT sensor also demonstrates the excellent selectivity toward the skin‐emitted trimethylamine (TMA) gas, with a theoretical limit of detection as low as 0.3 ppb. The work provides new insights into the preparation of the reliable skin‐like gas sensors and highlights the potential applications in the real‐time detection of skin gas and respiration gas for non‐invasive medical treatment and disease diagnosis.

## Graphical abstract

An intrinsically stretchable olfactory sensor is developed with excellent stretchability for seamlessly conforming onto human skin and with strain‐insensitive sensing response toward skin‐emitted gas at different stretching states. The high stability and stretchability provide a promising potential as an imperceptible real‐time monitor of skin/sweat emission and respiratory gas for rapid disease diagnosis and timely alarm.

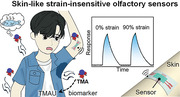

## 1 Introduction

Gases released from human skin or respiration, have been found as the biomarkers of diseases and physical conditions,^[^ ^1^, ^2^, ^3^, ^4^, ^5^, ^6^ ^]^ for example, acetone gas for diabetes,^[^ ^7^ ^]^ isoprene for non‐alcoholic fatty liver disease,^[^ ^8^ ^]^ ammonia for renal disease,^[^ ^9^ ^]^ TMA for trimethylaminuria (TMAU) and bacterial vaginitis (BV),^[^ ^10^, ^11^ ^]^ and nonanal for aging. However, the olfactory fatigue of the human nose, the low concentration of skin gas, or the intermittent gas excretion, possibly makes patients themselves difficult to perceive the released gas and causes missed and delayed diagnosis by the normal single medical examination. For example, TMAU easily leads to socially crippling disorders and suicidal tendencies, while most TMAU sufferers remain undiagnosed.^[^ ^10^, ^12^, ^13^, ^14^ ^]^ It may take years or even decades to successfully detect TMA before the diagnosis can be established. Therefore, it is urgent to develop wearable non‐invasive epidermal gas sensing systems for in‐time disease diagnosis and adjuvant medical therapy.

Intrinsically stretchable gas sensors, which are made of low‐modulus all‐stretchable electronic components, can be seamlessly integrated with human skin, allowing maximum proximity to gas release sources (skin or respiratory system), arbitrary wearable location (more privacy), and wearers high comfort (imperceptible wearing). Compared with the stretchable sensors based on geometric engineering strategies including surface wrinkles,^[^ ^15^, ^16^, ^17^ ^]^ porous/fiber network,^[^ ^18^, ^19^, ^20^ ^]^ electronic textiles,^[^ ^21^, ^22^, ^23^, ^24^, ^25^, ^26^ ^]^ and serpentine interconnections,^[^ ^27^, ^28^, ^29^, ^30^, ^31^ ^]^ etc., the intrinsically stretchable sensors also offer advantages such as high device density, good mechanical robustness, and skin compatibility,^[^ ^32^ ^]^ which make the intrinsically stretchable sensors very promising as the new‐generation wearable biomedical systems for the real‐time detection toward skin and respiration gas.

However, the intrinsically stretchable strategy inevitably requires the sensing/semiconductor components to suffer strain, which possibly significantly affects the device stability and gas diffusion‐reaction behavior, resulting in failure in accurate gas detection.^[^ ^20^, ^33^, ^34^ ^]^ The reported intrinsically stretchable gas sensors mainly are based on ion‐conducting materials, such as hydrogel/organohydrogel and ionotronic elastomers, which have shown excellent stretchability due to their crystal‐independent ionic conduction mode.^[^ ^35^, ^36^, ^37^, ^38^, ^39^, ^40^, ^41^ ^]^ Unfortunately, most of the ion‐conducting materials suffer from poor operation stability and severe baseline drift to humidity changes. As a result, the currently reported stretchable gas sensors mainly apply the geometric engineering strategy to achieve device stretchability, with the limited detection of gas species in atmospheric pollutants/environment such as NO_2,_ NH_3_, O_2_, relative humidity, etc. Therefore, the stretchable sensing material is still lacking, and it remains an enormous challenge to realize the strain‐insensitive highly stable intrinsically stretchable gas sensors for future skin‐like wearable electronics.

Herein, for the first time, the highly strain‐stable intrinsically stretchable gas sensors were fabricated based on the stretchable polymer semiconductors including PIDTBT, DPPT‐TT, and PCDTPT. The as‐prepared stretchable gas sensors can imperceptibly adhere on human skin and conform to different deformations such as bending, twisting, and stretching. They exhibit universal strain insensitivity to various gases such as TMA, NO_2_, and NH_3_. Furthermore, the intrinsically stretchable gas sensor exhibits excellent selectivity toward TMA, and a low limit of detection that is comparable to the average olfactory threshold of humans. This work provides new ideas for the preparation of reliable skin‐like gas sensors for real‐time monitoring of body‐emitted gases and shows the promising potential of the stretchable polymers for olfactory sensors to achieve in‐time disease diagnosis and real‐time adjuvant therapy.

## 2 Results and Discussion

**Figure 1:**
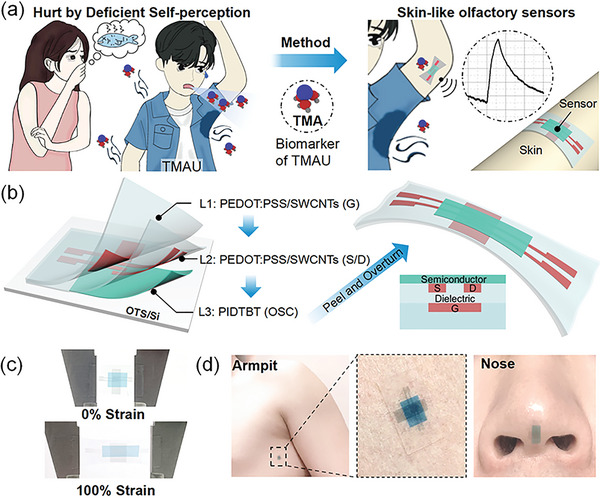
Electronic skin‐type artificial olfactory system monitoring the body gas for imperceptible health monitoring. a) Schematic illustrating the negative effect of a typical gaseous biomarker of the disease, TMA, and our resolution method with the skin‐like olfactory sensor. b) Fabrication and structure schematic of intrinsically stretchable transistor‐type gas sensors. c,d) The mechanical robustness and excellent conformability to human skin.

**Figure** **1**a shows the negative effect of a typical gaseous biomarker of the disease, TMA, and our resolution method with the skin‐like olfactory sensor. TMA is a volatile aliphatic tertiary amine. It is known for its characteristic odor of rotten fish and is released from the skin of TMAU patients. Olfactory fatigue and intermittent release of TMA make patients themselves generally difficult to perceive the presence of TMA or hardly identify the concentration of TMA but TMA can be detected by people around them, causing the patients severe social anxiety, social isolation, depression, and other mental diseases.^[^ ^10^, ^12^, ^13^, ^14^ ^]^ On the other hand, the concentration of TMA released by the TMAU patient is affected by the consumption of choline‐rich foods and drug treatment. Due to the lack of identification on the concentration of TMA, TMAU patients are usually plagued by excessive drug intake and strict diet control. Therefore, we developed a stretchable TMA sensor that can conform onto human skin, which not only can ensure comfort and privacy, but also can help patients and doctors to continuously track healthy processes, diagnose health conditions, assess disease risk, give timely alerts, and accurately monitor responses to medication.

Figure 1b illustrates the fabrication process and the schematic structure of the skin‐like sensor. The photolithographic source/drain and gate electrodes in PDMS were successively laminated onto the semiconductor layer, and then the whole device was peeled from the OTS/Si substrate to achieve the full‐stretchable device (experimental details are shown in Figure S1, Supporting Information). Polymer semiconductors as sensitive layers show the outstanding advantages in extremely low modulus and the creation of a transistor‐type sensor, with the purpose of both stretchability and amplified sensing signals. It is worth noting that the semiconductor layer is deliberately exposed on the top of the device, so that the semiconductor layer can easily contact with the gas molecules for gas sensing, which is different from our previously reported stretchable transistors where the semiconductor was sandwiched between source/drain and gate electrodes.^[^ ^42^, ^43^, ^44^ ^]^ In addition, the utilization of PEDOT:PSS&SWCNTs as an electrode material offers a notable advantage in terms of high‐precision patterning (down to 3 µm) through photolithography, surpassing conventional electrode materials such as carbon nanotube/metal nanowire networks, liquid metal, and doped elastomers. This characteristic lays a solid foundation for the development of highly integrated skin‐like gas sensor arrays in future applications.

Furthermore, Figure 1c,d demonstrates the mechanical robustness of the intrinsically stretchable gas sensor and its conformability on human skin. As shown in Figure 1c, the stretchable sensor was stretched by 100% strain without any failure observed. In Figure 1d, the sensor can be tightly attached to multiple parts of human skin, such as the armpits and nose. Such an intrinsically stretchable sensor shows the promising potential as an imperceptible real‐time monitor of skin/sweat emission and respiratory gas for rapid disease diagnosis and timely alarm, and also provides a chance to continuously detect harmful or toxic gases in an exposed environment.

**Figure 2:**
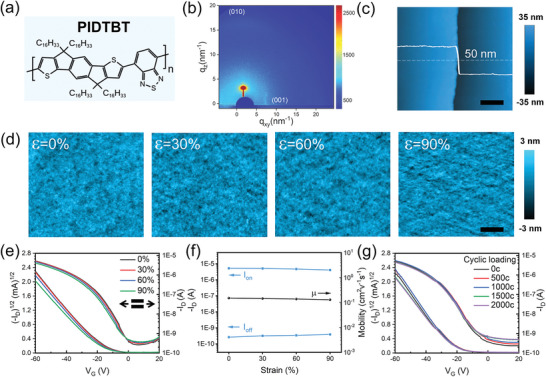
Chemical structure, morphologies, and electrical characterizations of the intrinsically stretchable gas sensor. a) Chemical structure, b) GIXRD pattern, and c) thickness of PIDTBT films. Scale bar: 800 nm. d) AFM images of the PIDTBT films illustrating the surface morphologies evolution under 0%, 30%, 60%, and 90% stretching strain. Scale bar: 300 nm. e,f) Typical transfer characteristics of the transistor‐type TMA sensor under stretching strains from 0% to 90%. g) The transfer characteristics of stretchable TMA sensor under released state after multiple stretching‐releasing cycles.

To obtain stable electrical performance under stretching conditions, it is necessary to apply the stretchable sensing material. Inorganic and organic small molecule films compose of continuous brittle crystalline regions, and hence are difficult to stretch. In contrast, the conjugated polymer semiconductor films have been known to possess a semi‐flexible chain‐type structure and a semicrystalline nature, thereby affording good flexibility and ductility.^[^ ^45^, ^46^, ^47^ ^]^ When stretched, the polymer semiconductors usually undergo fully reversible molecular rotation and chain elongation, and partially reversible molecular chain slippage in the amorphous dominated regions, which makes the polymers possess a larger crack onset strain compared with the inorganic and organic small‐molecular materials and even makes some polymers possess good stretchability. Based on this consideration, here, a near‐amorphous polymer semiconductor, c (PIDTBT), was selected as the sensing material. **Figure** **2**a shows its molecular structure that consists of a highly coplanar backbone and four bulky flexible alkyl side chains. Previous work has indicated that its bulky flexible alkyl side chains create great steric hindrance for high free volume, low Young's modulus, and near‐amorphous morphology, and the highly coplanar backbone provides a disorder‐free and crystal‐independent quasi‐1D charge transport mode, i.e., the strong intrachain transport mode.^[^ ^44^, ^48^, ^49^, ^50^, ^51^ ^]^ These advantages may allow the PIDTBT polymer chains to elongate or slide when stretched, resulting in efficient strain energy dissipation and strain‐stable charge transport process.

The grazing‐incidence X‐ray diffraction (GIXRD) pattern in Figure 2b shows the typical crystallinity and interchain organization at the molecular level of the PIDTBT film. The broad and diffused diffraction halo in the out‐of‐plane direction suggests the low crystallinity and the poor stacking orientation of the PIDTBT film, which was characterized as a “near amorphous” morphology for PIDTBT films in previous reports.^[^ ^51^ ^]^ In addition, a (010) peak at *q*_z_ direction of 22481.52 Å^−1^ was observed, corresponding to the calculated π–π stacking distance of 22484.1 Å by Bragg equation *(d = 2π/q*). This π–π spacing is larger than the extensively reported high‐performance conjugated polymers (for example, 3.5 Å of PCDTPT and 3.6 Å for DPPTT),^[^ ^48^, ^52^ ^]^ suggesting the large free volume of the PIDTBT backbone may originate from the loose and disorder molecular packing.^[^ ^44^, ^48^ ^]^ Figure 2c,d shows the thickness and surface morphology of the PIDTBT films characterized by atomic force microscopy. The unstretched polymer semiconductor film is only 50 nm thick and presents a uniform and dense morphology without any geometric engineering structures such as micropores or wrinkles, confirming its intrinsic stretchability. When the PIDTBT film is stretched, no cracks and obvious morphology changes are observed, and the film only shows a slight increase in roughness at 90% strain, illustrating the outstanding stretchability of PIDTBT.

Figure 2e,g explores the electrical properties of the TMA sensor under different tensile strains before the gas sensing test. The transfer curves of TMA sensors under different tensile strains are shown in Figure 2e. It can be seen that all TMA sensors show the typical characteristics of P‐type organic field effect transistors (OFETs) with the obvious carrier/signal modulation effect. When the tensile strain increases from 0% to 90%, both the drain current and the mobility of the transistors only show weak degradation. Such excellent electrical stability under the stretch strain fully demonstrates the advantages of the near‐amorphous structure and the intrachain‐dominated transport mode of the PIDTBT. Considering that human exercise is accompanied by the frequent stretching processes of the skin, the tensile strain toughness of the device was tested in Figure 2g. Since the tensile strain of most human skin is less than 30%, the robustness and tolerance of the device were investigated by 2000 cycles with 30% deformation cyclic stretching. All transfer curves show excellent coincidence and stability, which provides the necessary prerequisite for further gas sensing under stretching.

**Figure 3:**
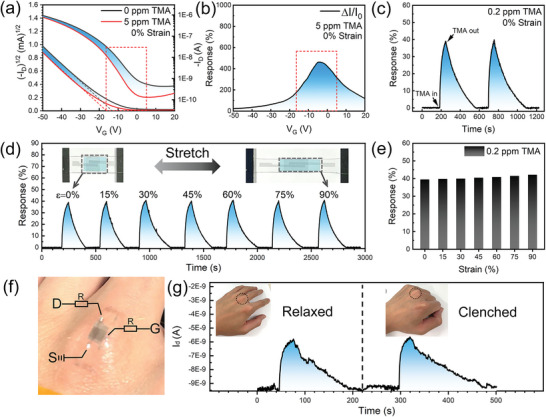
TMA sensing characterizations of the intrinsically stretchable sensor under different stretching strains at room temperature. a) Transfer characteristics of unstretched (0% strain) TMA sensor before and after 5 ppm TMA exposure. b) Response of unstretched gas sensor to TMA versus gate voltage. c) Two consecutive sensing cycles of unstretched gas sensors toward TMA. d,e) Dynamic TMA response of sensor under different stretching strains from 0% to 90%. f) A real photograph of the sensor adhered on the skin to in situ detected TMA. g) Real‐time TMA sensing of the sensor on relaxed and clenched hands to simulate real‐time detection of TMA gas released by the skin.

Based on the robust electrical properties of the PIDTBT film device against tensile strain, **Figure** **3** further demonstrates the gas‐sensing behavior of the device toward TMA and the effect of tensile strain on its gas‐sensing performance. First, the sensing performance of the unstretched device at 0% strain was investigated by the transfer curves at 0 and 5 ppm TMA, as shown in Figure 3a,c. With the injection of 5 ppm TMA, the *I*_d_ drops dramatically, originating from the strong electron‐donating properties of TMA. The TMA molecule has one lone pair of electrons and three electron‐pushing methyl groups, which make it behave as the electron donor and the shallow defect, and hence the TMA molecule can trap the holes and hinder the carrier transport in polymer semiconductors. Figure 3b shows the gate voltage‐modulated response value. When the negative *V*_g_ increases, the response value first increases and then decreases, and the maximum value appears in the subthreshold region (dashed rectangle in Figure 3b). This suggests that the gate voltage can effectively modulate and increase the response, showing the advantage of the field‐effect transistor configuration for sensors. According to the results in Figure 3b, we select the subthreshold region of OFET to further carry out the real‐time gas sensing test, with the fixed *V*_g_ at −6 V. Figure 3c shows two consecutive response/recovery curves. It can be seen that the response increases rapidly and then slowly decreases with the discharge of TMA. The response value of the second cycle is the same as the first cycle. The good recoverability of the current and the high repeatability of the response value indicate that the TMA sensor can effectively overcome the problem of human olfactory fatigue.

Figure 3d,g systematically investigated the effect of different tensile strains on the TMA sensing performance. The sensing performance toward 200 ppb TMA was tested at tensile strain (*ε*) of 0%, 15%, 30%, 45%, 60%, 75%, and 90%, respectively. It is amazing that the response remains almost unchanged at the fixed TMA concentration even when the sensor is stretched up to 90%. Such strain‐insensitive sensing performance brings outstanding advantages in high signal reliability, elimination of additional calibration equipment, reduced cost, and improved integration. For further practical applications, we attached the sensor to the skin of the finger joint and performed the gas sensing tests to simulate the process of in situ detection of TMA released from the skin (Figure 3f). As shown in Figure 3g, the palm of the hand, respectively, remained relaxed and clenched to change the strain of the device, and the corresponding electrical signals were recorded. It can be clearly seen that the sensor can tightly adhere on the skin and conformally deform with the movement of the skin, and the sensing performance remains good stability, demonstrating the great application potential in imperceptible non‐invasive medicine and health monitoring.

In Figure 3, a surprising result is the strain‐insensitive gas response in the PIDTBT film sensor even at the tensile strain of 90%. Our previous work has shown that the weak strain only at 0.7% can significantly increase the response value of a single‐crystal small molecule semiconductor device by one order of magnitude.^[^ ^33^ ^]^ It is believed that the effects of tensile strain on the molecular arrangement and gas diffusion processes are significantly different for polymers and small molecules. In a typical sensing process, some of the gas molecules are adsorbed onto the surface of the semiconductor and react with the exposed active sites, while some possibly can diffuse into the semiconductor, resulting in dramatic changes of the current. Generally, the gas diffusion‐reaction behavior mainly includes three modes: bulk Poiseuille flow (*d*_material_>*λ*>*d*_gas_), Knudsen diffusion (*λ> d*_material_ *>d_gas_*), and solid‐state diffusion *(λ>d*_gas_ *>d*_material_), where *d*_material_ the pore size or molecule distance of materials, *λ* is the mean free path of the gas molecule, and *d*_gas_ is the diameter of the gas molecule.^[^ ^53^, ^54^, ^55^ ^]^ It is well known that the small‐molecule organic single crystals, with the individual small molecules as a unit, usually present the long‐range order and compact molecular packing typically at ≈3–4 Å, which is similar to and even smaller than the diameter of gas molecules (3–6 Å). Therefore, the small‐molecule single crystal at the unstretched state mainly follows the solid‐state diffusion and the sensing reactions mainly occur on the material surface. When it is stretched, the tensile stress increases the molecular spacing and makes the diffusion mode possibly transform into Knudsen diffusion. Hence, previous work indicated that tensile stress increases the depth of gas diffusion or increases the specific surface area.^[^ ^20^, ^33^ ^]^ And the reaction occurs both on the surface and inside of the small‐molecular single crystal, resulting in the dramatically changed gas response as shown in the previous report.

In contrast, the polymers, with the long and flexible molecule chains as units and with the ordered crystalline regions embedded in disordered amorphous regions, present far larger packing distances, and a more complex crystal structure. For example, previous GIXRD patterns have shown that polymer semiconductors have ultra‐large intermolecular spacings of even a few nanometers along the lamella stack direction in small crystalline regions, for example, 16.91 Å for P3HT,^[^ ^56^ ^]^ 25 Å for PCDTPT,^[^ ^52^ ^]^ 26 Å for PIDTBT,^[^ ^49^ ^]^ 26.49 Å for PDPPTT,^[^ ^57^ ^]^ 27.8 Å for PBDT‐3T,^[^ ^58^ ^]^ 32.2 Å for p(g7NCnN),^[^ ^59^ ^]^ 34.06 Å for CSi‐PBIBDF,^[^ ^60^ ^]^ 43.32 Å for 12PTTP12.^[^ ^61^ ^]^ Moreover, there may be a looser molecular arrangement in the amorphous region due to its disordered nature. Such intermolecular spacings exceed the diameter of typical gas molecules, such as TMA at 5 Å, while remaining less than the mean free path of the majority of gas molecules, which typically ranges several tens of nanometers under ambient pressure and temperature conditions. This allows gas molecules to move inside the polymer film by Knudsen diffusion.^[^ ^53^, ^54^, ^62^ ^]^ That is, in addition to surface reactions, gas molecules can also undergo a diffusion‐reaction coupling process into the interior of the film, causing a sufficient gas‐sensing reaction even in unstretched polymer films.

Different from the small‐molecular single crystals, the polymer films generally exhibit the distinctive strain dissipation mechanism when stretched, due to their lubrication effect of side chains, the flexibility of long main chains, and the inhomogeneous hardness distribution caused by incomplete crystallization.^[^ ^46^, ^63^ ^]^ Previous wide/small‐angle X‐ray diffraction patterns, polarized UV–vis optical absorption spectroscopies, and coarse‐grained molecular dynamics simulations have observed the changed diffraction peak intensities along the different crystal directions, and the increased dichroic ratios and end‐to‐end lengths upon film deformation.^[^ ^44^, ^48^, ^63^, ^64^ ^]^ The tensile strain is believed to be usually dissipated by the conformational change and the slippage/alignment of molecular chains toward the parallel‐to‐strain direction, and the rotation of the crystallites.^[^ ^48^, ^49^, ^63^, ^64^, ^65^ ^]^ This means that the polymer films neither significantly increase the molecular distance nor easily disrupt the crystallites like the small‐molecule single crystals and the traditional metal oxide materials. Therefore, the stretched polymer film still obeys the Knudsen diffusion model and undergoes a sufficient gas‐sensing response both on the film surface and inside the film, resulting in the stain‐insensitive gas‐sensing response at different stretched states.

**Figure 4:**
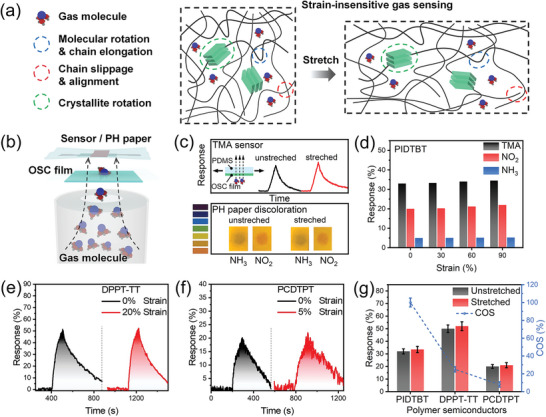
Mechanism and universality of strain‐insensitive gas sensing properties. a) The schematic of the tensile strain‐evolved deformation and strain‐insensitive gas diffusion mechanism. b) Schematic diagram of gas permeability experiment. c) TMA sensor response curve and H test paper discoloration intuitively demonstrate the permeability of PIDTBT to TMA, NO_2_, and NH_3_, respectively. d) The universality of strain‐insensitive sensing to different gases. e‐g) TMA sensor response curve based on DPPT‐TT and PCDTPT semiconductor films, and comparison with PIDTBT films toward strain insensitivity and COS.

Further experiments intuitively confirm the large molecular chain spacing and the Knudsen diffusion in the polymer thin film by revealing the permeability of gas molecules in polymer thin film. As shown in **Figure** **4**b, a container with one round outlet is filled with TMA, NH_3_, and NO_2_, respectively. The outlet is sealed by a suspended unstretched PIDTBT film or another stretched film. The sensor and pH paper were used, respectively, to monitor the gas permeability of the PIDTBT film. The detailed experimental procedure and discussion are presented in the Supporting Information. As shown in Figure 4c, when the TMA is sealed in the container, the sensors exhibit an almost unchanged response when the container is sealed by the unstretched and stretched PIDTBT film, respectively, which directly proves that TMA molecular can penetrate the PIDTBT film through free diffusion motion. When NH_3_ and NO_2_ were respectively sealed in the container by PIDTBT film, and then the pH paper was placed above the film for 60 s, the pH test strips showed obvious and same discoloration, confirming the penetration capability of NO_2_ and NH_3_ in PIDTBT film regardless of the stretched state of film. Figure 4d further shows the response of the stretched PIDTBT sensor toward different gases including TMA, NH_3,_ and NO_2_. When the stretching strain changes from 0% to 90%, excellent stain‐insensitive gas sensing properties also can be observed, which demonstrates that the strain‐insensitive gas‐sensing behavior of polymers should be universal to different gases.

Further, to confirm the universality of the strain‐insensitive gas sensing properties, we chose the other two higher‐crystallinity polymer semiconductors compared with the near‐amorphous PIDTBT, the semi‐crystalline diketo‐pyrrolo‐pyrrole bithiophene thienothiophene (DPPT‐TT) and high‐crystallinity poly[4‐(4,4‐dihexadecyl‐4H‐cyclopenta[1,2‐b:5,4‐b′]dithiophen‐2‐yl)‐alt‐[1,2,5]thiad‐iazolo [3,4‐c] pyridine] (PCDTPT).^[^ ^48^, ^52^, ^57^ ^]^ Prior to gas response testing, the crack onset strain was measured to be ≈100%, 20%, and 5% for PIDTBT, DPPT‐TT, and PCDTPT films, respectively. Then their gas‐sensing properties at near crack onset strain were compared with those at unstretched states. As shown in Figures 3d and 4e,g, all of the polymer‐based sensors present a strain‐insensitive gas response when stretched. These results indicate the universality of the strain‐stable gas sensing behavior for polymer semiconductors, and this behavior is independent of the crystallinity of the polymer semiconductor. However, the high‐crystallinity polymers usually possess a lower crack onset strain, which limits the tensile strain that the device can withstand and decreases the robustness of the gas sensor. When the applied tensile strain exceeds the crack onset strain, the crystal fracture occurs and the cracks appear, which will seriously damage the device resulting in performance degradation and the device and even failed operation. Therefore, to achieve a highly strain‐stable stretchable gas sensor with a wide range of stress endurance limits, it is preferred to choose polymers with poor symmetry, rich conformation, and large side chains.

**Figure 5:**
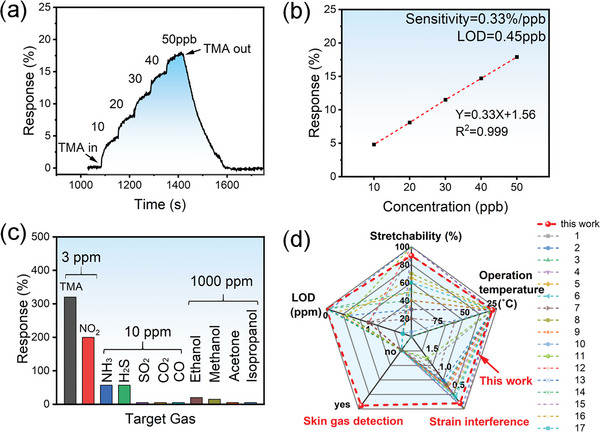
Linearity, LOD, selectivity, and comparison with the reported literature of stretchable gas sensors. a,b) Response of sensor to TMA at range from 10 to 50 ppb. c) Selectivity of intrinsically stretchable gas sensors toward different gases. d) Comparison of comprehensive performance with the reported stretchable gas sensors.

Considering the low concentration of skin gases, the limit of detection of the sensor toward the TMA gas was further explored in **Figure** **5**a,b. The sensor shows a discernable current change even toward 10 ppb TMA, and the response curve presents excellent linearity in the concentration range of 10–50 ppb. The sensitivity calculated from the slope of the response versus concentration is 1% ppb^−1^. In accordance with the International Union of Pure and Applied Chemistry (IUPAC) procedure, the limit of detection (LOD) of the sensor can be estimated by:

(1) LOD=3×RMS/S

where RMS is the standard root‐mean‐square deviation for the noise of the sensor and *S* is sensitivity.^[^ ^66^ ^]^ The RMS was calculated from 100 data points of the current baseline before the TMA gas was introduced. The calculated RMS is 0.1%. Accordingly, the theoretical LOD is 0.3 ppb, which is at the same level as the average olfactory threshold of humans toward TMA. Notably, the stretchable olfactory sensor presents good conformability. It can tightly adhere onto the skin surfaces near those areas of exhalation or heavy sweating, including the nose, armpits, palms, etc. which provides the outstanding advantage in capturing higher‐concentration skin gas more quickly compared with the human olfactory system, since the skin gas needs to diffuse around the human nose to be perceived. Therefore, the designed sensor presents the ability to monitor the level of TMA released by the skin in real time, which can effectively help the patients to carry out drug adjunctive therapy, so as to successfully control the concentration of TMA under the perceivable level by nearby people, help them to alleviate and even overcome the social anxiety problem. Figure 5c explores the selectivity of the PIDTBT sensor for different gases. It can be seen that the response value of the sensor to 3 ppm TMA is 300%, which is obviously higher than that of NO_2_, NH_3_, H_2_S, SO_2_, NO, and CO at the same concentration_,_ and 1000 ppm ethanol, methanol, acetone, isopropanol, and other organic volatile gases. This means that the sensor has excellent selectivity for TMA detection and anti‐interference capability. Figure 5d shows a comparison of our sensor with the reported stretchable gas sensor in terms of selectivity, operating temperature, stretchability, the limit of detection, and strain interference. The details of the literature comparison and the definition of strain interference are shown in Table S1 (Supporting Information). Obviously, it has more prominent advantages in LOD, strain stability, and innovative applications for skin gas detection, which will provide new ideas for the preparation of highly reliable electronic skin‐type sensors and important applications in non‐invasive disease diagnosis and imperceptible health monitoring.

## 3 Conclusion

In summary, we developed a novel strain‐insensitive intrinsically stretchable gas sensor based on stretchable polymer semiconductors. The universality of the strain‐insensitive gas‐sensing behavior of the stretchable polymers is confirmed by applying the polymers with different degrees of crystallinity as the sensitive materials. It is found that the gas molecules can permeate the polymer films, which is ascribed to the ultra‐large molecular spacing of the polymers that allow gas molecules to diffuse into the film interior whether the polymer films are stretched or not. The strain‐stable gas sensing behavior of the stretchable polymer‐based devices, combined with their imperceptible adherence capability onto the human skin, makes the all‐stretchable polymer sensors present promising potential in skin‐like electronics. In addition, the intrinsically stretchable gas sensor exhibits good selectivity toward TMA gases and the limit of detection comparable to the human olfactory threshold, which allows the sensor to achieve in‐time disease diagnosis and real‐time adjuvant therapy of diseases associated with skin gas. Our work provides meaningful guidance for the design of reliable intrinsically stretchable gas sensors and shows the potential application of the sensors in the real‐time detection of skin gas in non‐invasive disease diagnosis and imperceptible health monitoring.

## 4 Experimental Section

### Materials

PIDTBT with molecular weights (Mw) of 310 kDa and DPPT‐TT with Mw of 194 kDa were purchased from Derthon Optoelectronic Materials Co. Ltd. PCDTPT with Mw of 79 kDa was purchased from 1‐material. PEDOT: PSS aqueous solution (Clevios PH1000) with a solid concentration of 1.0–1.3 wt.% was purchased from Heraeus. Ethylene glycol (EG) and surface‐active agent (Capstone FS‐30) were purchased from Sigma–Aldrich and DuPont, respectively. PDMS (Sylgard 184) was purchased from Dow Corning. The SWCNTs aqueous dispersion with an initial concentration of 0.22 mg mL^−1^ and a purity of >95 wt.% was purchased from Chengdu Organic Chemicals Co. Ltd., Chinese Academy of Sciences.

### Fabrication and Characterization of Intrinsically Stretchable Gas Sensors

The detailed process schematics of the intrinsically stretchable bottom‐gate/bottom‐contact (BGBC) FET‐type gas sensors are shown in Figure S1 (Supporting Information). First, the cleaned Si substrate was first surface‐modified with a monolayer molecule for subsequent exfoliation of the thin film, by oxygen plasma treatment followed by immersion into an octadecyltrimethoxysilane (OTS) solution (OTS: heptane = 1:1000, v/v). Then, gate electrodes embedded in the support layer (L1), source/drain electrodes with a length–width ratio of 40:1 embedded in the dielectric layer (L2), and the semiconductor layer (L3) were prepared on the three OTS‐modified Si substrates, respectively. In detail, the conductive components of both L1 and L2 use the patterned PEDOT: PSS/SWCNTs hybrid electrodes fabricated by the conventional photolithography process, as we reported previously.^[^ ^67^ ^]^ Then the PDMS solution (cross‐linking ratio of 10:1) was spin‐coated onto the gate electrode as a support layer of L1, and the diluted PDMS solution (PDMS: hexane = 1:6, v/v) was spin‐coated onto the source/drain electrode as a dielectric layer of L2. L3 can be obtained by dissolving PIDTBT in chloroform (5 mg mL^−1^) and spin‐coating on OTS‐modified Si (1500 rpm, 60 s). To maintain the higher amorphous morphology, the spin‐coated PIDTBT films were not further annealed. Finally, The BGBC FET‐type gas sensor can be assembled by laminating L1 onto L2, then peeling off L3 with L1&L2, and finally flipping L1&L2&L3 over.

To obtain the stretched PIDTBT films for the characterizations, we transferred the PIDTBT films onto 0.3 mm thick PDMS films (cross‐linking ratio of 10:1). PIDTBT films on PDMS were stretched using a custom‐built stretching station. The macroscopic and microscopic morphology changes of stretched PIDTBT films were determined with an optical microscope and AFM measurements. Optical microscopy investigations were performed with an Olympus BX51 microscope and a Keyence VHX‐5000 (Keyence, Japan). AFM measurements were performed in the air with a Bruker Dimension Icon instrument (Bruker, Germany). GIXRD data were obtained at 1W1A, Beijing Synchrotron Radiation Facility (l = 1.5418 Å).

### Electrical and Gas‐sensing Properties Measurements

Electrical properties and sensing characteristics were investigated using a homemade analysis system consisting of a flow‐monitoring system, a custom‐built stretching station, a homemade testing chamber, and the Keithley 4200 SCS station. The gas distribution process includes two parts. Inorganic toxic gas (NO_2_, NO, SO_2_, H_2_S, NH_3_, and CO) adopted dynamic gas distribution, which was accurately controlled and mixed with dry air (background gas) using multiple gas flow meters (500 SCCM, Beijing Sevenstar Electronics Company, CS200). Volatile organic compounds (TMA, ethanol, acetone, methanol, and isopropanol) adopted the static volumetric method. The gas‐sensing properties of the sensors were measured using a static volumetric method. The sensors were tested in a sealed chamber (7 L). When the current of the sensors was stable, the calculated target gas was injected into the chamber by an injector, which will be quickly and uniformly distributed in the test chamber under the drive of the fan. For the target gases obtained from liquid, the concentration of target gas was calculated by the following formula (2):

(2) $$C=\frac{22.4\rho dV_{1}}{MV_{2}}$$

where *C* (ppm) is the target gas concentration, *ρ* (g mL^−1^) is the density of the liquid, *d* is the purity of the liquid, *V_1_* (L) is the volume of the liquid, *V*_2_ (L) is the volume of the sealed chamber, and *M* (g mol^−1^) is the molecular weight of the liquid. During the recovery phase, a high‐flow vacuum pump (30 SLM) was used to exhaust the target gas and replenish the background gas. The response value of the gas sensor is calculated according to the following formula (3):

(3) $$R_{\mathrm{oxidizing}}=\frac{I_{g}-I_{0}}{I_{0}}\;\times 100\%,\;\;R_{\mathrm{reducing}}=\frac{I_{0}-I_{g}}{I_{g}}\;\times 100\%$$

(1) where *R*_oxidizing_ and *R*_reducing_ are the response of gas sensors toward oxidizing and reducing gases. *I*_0_ and *I*_g_ are the current in the air and the testing gas atmosphere, respectively. The sensitivity of the gas sensor is calculated from the ratio of response to the concentration of testing gases.

Field‐effect mobilities (*µ*) were calculated in the saturation regime using the following formula (4):

(4) $$\mu \;=\frac{2L}{WC_{i}}\;{\left(\frac{\partial \sqrt{I_{\mathrm{DS}}}}{\partial V_{G}}\right)}^{2}$$

where *W* and *L* stand for the width and length of the channel, respectively, *C*_i_ denotes the capacitance per unit area of the dielectric layer, *I*_DS_ is the source‐drain current, and *V*_G_ represents the gate voltage.

## Conflict of Interest

The authors declare no conflict of interest.

## Supporting information

Supporting Information

## Data Availability

The data that support the findings of this study are available from the corresponding author upon reasonable request.

## References

[1] K. J.‐S. Seong‐Yong , L. Jong‐Heun , Adv. Mater. 2020, 32, 2002075.

[2] I. Mogilnicka , P. Bogucki , M. Ufnal , Int. J. Mol. Sci. 2020, 21, 2886. 32326126 10.3390/ijms21082886 PMC7215946

[3] A. Annerino , P. P. Gouma , Sensors 2021, 21, 7554. 34833630 10.3390/s21227554 PMC8618486

[4] Y. Cheng , R. Yang , J.‐P. Zheng , Z. L. Wang , P. Xiong , Mater. Chem. Phys. 2012, 137, 372.

[5] S. K. Jha , Rev. Anal. Chem. 2017, 36, 20160028.

[6] A. Agapiou , A. Amann , P. Mochalski , M. Statheropoulos , C. L. P. Thomas , TrAC, Trends Anal. Chem. 2015, 66, 158.

[7] H. S. Koo , D. Michaelson , K. Teel , D.‐J. Kim , H. Park , M. Park , G. Stylios , Int. J. Cloth. Sci. Technol. 2016, 28, 10.1108/IJCST-10-2015-0113.

[8] N. Alkhouri , F. Cikach , K. Eng , J. Moses , N. Patel , C. Yan , I. Hanouneh , D. Grove , R. Lopez , R. Dweik , Eur. J. Gastroenterol. Hepatol. 2014, 26, 82. 24284369 10.1097/MEG.0b013e3283650669

[9] C. Lou , C. Yang , W. Zheng , X. Liu , J. Zhang , Sens. Actuators, B 2021, 329, 129218.

[10] D. Roddy , P. McCarthy , D. Nerney , J. Mulligan‐Rabbitt , E. Smith , E. P. Treacy , JIMD Rep. 2021, 57, 67. 33473342 10.1002/jmd2.12170 PMC7802621

[11] D. L. Cruden , R. P. Galask , Microb. Ecol. Heal. Dis. 2009, 1, 95.

[12] F. Patel , Y. M. Tu , S. Fernandes , A. Chapas , JAAD Case Rep. 2019, 5, 915. 31646163 10.1016/j.jdcr.2019.07.018 PMC6804454

[13] N. Ramos , C. Wystrach , M. Bolton , J. Shaywitz , W. W. IsHak , J. Nerv. Ment. Dis. 2013, 201, 537. 23719328 10.1097/NMD.0b013e31829482fd

[14] J. Christodoulou , J. Paediatr. Child Health 2012, 48, E153. 21276117 10.1111/j.1440-1754.2010.01978.x

[15] Z. Song , Z. Huang , J. Liu , Z. Hu , J. Zhang , G. Zhang , F. Yi , S. Jiang , J. Lian , J. Yan , J. Zang , H. Liu , ACS Sens. 2018, 3, 1048. 29737152 10.1021/acssensors.8b00263

[16] H. Yan , M. Zhong , Z. Lv , P. Wan , Small 2017, 13, 1701697. 10.1002/smll.201701697 28895272

[17] H. Guo , C. Lan , Z. Zhou , P. Sun , D. Wei , C. Li , Nanoscale 2017, 9, 6246. 28466937 10.1039/c7nr01016h

[18] M. L. Jin , S. Park , J. S. Kim , S. H. Kwon , S. Zhang , M. S. Yoo , S. Jang , H. J. Koh , S. Y. Cho , S. Y. Kim , C. W. Ahn , K. Cho , S. G. Lee , D. H. Kim , H. T. Jung , Adv. Mater. 2018, 30, 1706851. 10.1002/adma.201706851 29603454

[19] C. Liu , M. Wu , L. Gao , H. Liu , J. Yu , Sens. Actuators, B 2022, 371, 132540.

[20] B. Wang , A. Thukral , Z. Xie , L. Liu , X. Zhang , W. Huang , X. Yu , C. Yu , T. J. Marks , A. Facchetti , Nat. Commun. 2020, 11, 2405. 32415064 10.1038/s41467-020-16268-8 PMC7229221

[21] Y. Tang , Y. Xu , J. Yang , Y. Song , F. Yin , W. Yuan , Sens. Actuators, B 2021, 346, 130500.

[22] L. T. Duy , T. Q. Trung , A. Hanif , S. Siddiqui , E. Roh , W. Lee , N.‐E. Lee , 2D Mater. 2017, 4, 025062.

[23] L. Sang Won , L. Wonseok , K. Insu , L. Dongtak , P. Dongsung , K. Woong , P. Jinsung , L. Jeong Hoon , L. Gyudo , Y. Dae Sung , ACS Sens. 2020, 6, 777.33253539

[24] C. Won , S. Lee , H. H. Jung , J. Woo , K. Yoon , J. Lee , C. Kwon , M. Lee , H. Han , Y. Mei , K.‐I. Jang , T. Lee , ACS Appl. Mater. Interfaces 2020, 12, 45243. 32893618 10.1021/acsami.0c10460

[25] D. Thanh Hoang Phuong , T. Qui Thanh Hoai , S. Adem , H. Nguyen Thuy , Y. Woochul , N. Jin‐Seo , ACS Sens. 2020, 5, 2255.32597174

[26] W. Li , R. Chen , W. Qi , L. Cai , Y. Sun , M. Sun , C. Li , X. Yang , L. Xiang , D. Xie , T. Ren , ACS Sens. 2019, 4, 2809. 31566369 10.1021/acssensors.9b01509

[27] Y. Li , Y. Ning , Z. Jia , C. Zheng , Y. Xinyang , Z. Xueyi , Z. Hongli , C. Huanyu , J. Mater. Chem. A 2020, 8, 6487.

[28] Md. A. Islam , H. Li , S. Moon , S. S. Han , H.‐S. Chung , J. Ma , C. Yoo , T.‐ Ko , K. H. Oh , Y. J. Jung , Y. Jung , ACS Appl. Mater. Interfaces 2020, 12, 53174. 33180481 10.1021/acsami.0c17540

[29] Y. Ning , C. Zheng , L. Han , Y. Li , Z. Jia , Z. Xiaoqi , C. Yong , L. Zhendong , Z. Hongli , C. Huanyu , Mater. Today Phys. 2020, 15, 100265.

[30] J. Yun , Y. Lim , G. N. Jang , D. Kim , S.‐J. Lee , H. Park , S. Y. Hong , G. Lee , G. Zi , J. S. Ha , Nano Energy 2016, 19, 401.

[31] J. Park , J. Kim , K. Kim , S. Y. Kim , W. H. Cheong , K. Park , J. H. Song , G. Namgoong , J. J. Kim , J. Heo , F. Bien , J. U. Park , Nanoscale 2016, 8, 10591. 27166976 10.1039/c6nr01468b

[32] W. C. Wang , S. H. Wang , R. Rastak , Y. Ochiai , S. M. Niu , Y. W. Jiang , P. K. Arunachala , Y. Zheng , J. Xu , N. Matsuhisa , X. Z. Yan , S. K. Kwon , M. Miyakawa , Z. T. Zhang , R. Ning , A. M. Foudeh , Y. Yun , C. Linder , J. B. H. Tok , Z. N. Bao , Nat. Electron. 2021, 4, 143.

[33] K. Tang , Z. Song , Q. Tang , H. Tian , Y. Tong , Y. Liu , IEEE Electron Device Lett. 2018, 39, 119.

[34] G. Namgung , Q. T. H. Ta , W. Yang , J. S. Noh , ACS Appl. Mater. Interfaces 2019, 11, 1411. 30525384 10.1021/acsami.8b17336

[35] Y. Liang , Z. Wu , Y. Wei , Q. Ding , M. Zilberman , K. Tao , X. Xie , J. Wu , Nanomicro Lett 2022, 14, 52. 35092489 10.1007/s40820-021-00787-0 PMC8800976

[36] Y. Wei , H. Wang , Q. Ding , Z. Wu , H. Zhang , K. Tao , X. Xie , J. Wu , Mater. Horiz. 2022, 9, 1921. 35535754 10.1039/d2mh00284a

[37] Y. Liang , Q. Ding , H. Wang , Z. Wu , J. Li , Z. Li , K. Tao , X. Gui , J. Wu , Nanomicro Lett 2022, 14, 183. 36094761 10.1007/s40820-022-00934-1 PMC9468213

[38] Z. Wu , Q. Ding , Z. Li , Z. Zhou , L. Luo , K. Tao , X. Xie , J. Wu , Sci. China Mater. 2022, 65, 2540. 35600911 10.1007/s40843-021-2022-1 PMC9109751

[39] W. Zixuan , Y. Xing , W. Jin , ACS Appl. Mater. Interfaces 2021, 13, 2128. 33405508 10.1021/acsami.0c21841

[40] Z. Wu , L. Rong , J. Yang , Y. Wei , K. Tao , Y. Zhou , B. R. Yang , X. Xie , J. Wu , Small 2021, 17, 2104997. 10.1002/smll.202104997 34672085

[41] J. Wu , Z. Wu , W. Huang , X. Yang , Y. Liang , K. Tao , B. R. Yang , W. Shi , X. Xie , ACS Appl. Mater. Interfaces 2020, 12, 52070. 33147020 10.1021/acsami.0c17669

[42] X. Wang , P. Zhao , Y. Tong , S. Guo , G. Zhao , M. Zhang , H. Yu , X. Zhao , Q. Tang , Y. Liu , Adv. Mater. Technol. 2022, 7, 2200660.

[43] S. Guo , Y. Tong , X. Wang , M. Zhang , H. Yu , H. Ren , Q. Tang , G. Lu , Y. Liu , Adv. Electron. Mater. 2022, 2200438, 10.1002/aelm.202200438.

[44] H. Ren , J. Zhang , Y. Tong , J. Zhang , X. Zhao , N. Cui , Y. Li , X. Ye , Q. Tang , Y. Liu , J. Mater. Chem. C 2020, 8, 15646.

[45] H.‐C. Tien , Y.‐W. Huang , Y.‐C. Chiu , Y.‐H. Cheng , C.‐C. Chueh , W.‐Y. Lee , J. Mater. Chem. C 2021, 9, 2660.

[46] Y. Zheng , S. Zhang , J. B. Tok , Z. Bao , J. Am. Chem. Soc. 2022, 144, 4699. 35262336 10.1021/jacs.2c00072

[47] Y. Yao , H. Dong , W. Hu , Adv. Mater. 2016, 28, 4513. 26634645 10.1002/adma.201503007

[48] Y. Zheng , G. J. N. Wang , J. Kang , M. Nikolka , H. C. Wu , H. Tran , S. Zhang , H. Yan , H. Chen , P. Y. Yuen , J. Mun , R. H. Dauskardt , I. McCulloch , J. B. H. Tok , X. Gu , Z. Bao , Adv. Funct. Mater. 2019, 29, 1905340.

[49] X. Zhang , H. Bronstein , A. J. Kronemeijer , J. Smith , Y. Kim , R. J. Kline , L. J. Richter , T. D. Anthopoulos , H. Sirringhaus , K. Song , M. Heeney , W. Zhang , I. McCulloch , D. M. DeLongchamp , Nat. Commun. 2013, 4, 2238. 23900027 10.1038/ncomms3238

[50] B. Zhao , D. Pei , Y. Jiang , Z. Wang , C. An , Y. Deng , Z. Ma , Y. Han , Y. Geng , Macromolecules 2021, 54, 9896.

[51] D. Venkateshvaran , M. Nikolka , A. Sadhanala , V. Lemaur , M. Zelazny , M. Kepa , M. Hurhangee , A. J. Kronemeijer , V. Pecunia , I. Nasrallah , I. Romanov , K. Broch , I. McCulloch , D. Emin , Y. Olivier , J. Cornil , D. Beljonne , H. Sirringhaus , Nature 2014, 515, 384. 25383522 10.1038/nature13854

[52] S. N. Patel , G. M. Su , C. Luo , M. Wang , L. A. Perez , D. A. Fischer , D. Prendergast , G. C. Bazan , A. J. Heeger , M. L. Chabinyc , E. J. Kramer , Macromolecules 2015, 48, 6606.

[53] T. Li , Y. Wu , J. Huang , S. Zhang , Sens. Actuators, B 2017, 243, 566.

[54] C. Yuan , J. Ma , Y. Zou , G. Li , H. Xu , V. V. Sysoev , X. Cheng , Y. Deng , Adv. Sci. 2022, 9, 2203594. 10.1002/advs.202203594 PMC9685467 36116122

[55] G. Sakai , N. Matsunaga , K. Shimanoe , N. Yamazoe , Sens. Actuators, B 2001, 80, 125.

[56] H. J. Cheon , S. Y. Shin , V. V. Tran , B. Park , H. Yoon , M. Chang , Chem. Eng. J. 2021, 425, 131424.

[57] D. Liu , J. Mun , G. Chen , N. J. Schuster , W. Wang , Y. Zheng , S. Nikzad , J. C. Lai , Y. Wu , D. Zhong , Y. Lin , Y. Lei , Y. Chen , S. Gam , J. W. Chung , Y. Yun , J. B. Tok , Z. Bao , J. Am. Chem. Soc. 2021, 143, 11679. 34284578 10.1021/jacs.1c04984

[58] Y.‐W. Huang , Y.‐C. Lin , H.‐C. Yen , C.‐K. Chen , W.‐Y. Lee , W.‐C. Chen , C.‐C. Chueh , Chem. Mater. 2020, 32, 7370.

[59] A. Marks , X. Chen , R. Wu , R. B. Rashid , W. Jin , B. D. Paulsen , M. Moser , X. Ji , S. Griggs , D. Meli , X. Wu , H. Bristow , J. Strzalka , N. Gasparini , G. Costantini , S. Fabiano , J. Rivnay , I. McCulloch , J. Am. Chem. Soc. 2022, 144, 4642. 35257589 10.1021/jacs.2c00735 PMC9084553

[60] Y. Ding , Y. Yuan , N. Wu , X. Wang , G. Zhang , L. Qiu , Macromolecules 2021, 54, 8849.

[61] D. Liu , Y. Chu , X. Wu , J. Huang , Sci. China Mater. 2017, 60, 977.

[62] L. Liu , W. Xiong , L. Cui , Z. Xue , C. Huang , Q. Song , W. Bai , Y. Peng , X. Chen , K. Liu , S. Zhang , L. Wen , Y. Che , T. Wang , Angew. Chem., Int. Ed. 2020, 59, 15953. 10.1002/anie.202006408 32519404

[63] S. Zhang , A. Alesadi , G. T. Mason , K. L. Chen , G. Freychet , L. Galuska , Y. H. Cheng , P. B. J. St. Onge , M. U. Ocheje , G. Ma , Z. Qian , S. Dhakal , Z. Ahmad , C. Wang , Y. C. Chiu , S. Rondeau‐Gagné , W. Xia , X. Gu , Adv. Funct. Mater. 2021, 31, 2100161.

[64] B. O'Connor , R. J. Kline , B. R. Conrad , L. J. Richter , D. Gundlach , M. F. Toney , D. M. DeLongchamp , Adv. Funct. Mater. 2011, 21, 3697.

[65] R. Noriega , J. Rivnay , K. Vandewal , F. P. Koch , N. Stingelin , P. Smith , M. F. Toney , A. Salleo , Nat. Mater. 2013, 12, 1038. 23913173 10.1038/nmat3722

[66] C. Li , P. G. Choi , Y. Masuda , Adv. Sci. 2022, 2202442. 10.1002/advs.202202442 PMC9507369 35839470

[67] N. Cui , Q. Tang , H. Ren , X. Zhao , Y. Tong , Y. Liu , J. Mater. Chem. C 2019, 7, 5385.

