# Supplementary material for: Highly Strain‐Stable Intrinsically Stretchable Olfactory Sensors for Imperceptible Health Monitoring
Source: Adv Sci (Weinh). 2023 Aug 23;10(29):2302974. doi: 10.1002/advs.202302974 (PMC10582427; doi:10.1002/advs.202302974)
Supplement: Supplementary file 1 — Supporting Information [file ADVS-10-2302974-s001.pdf]

ADVANCED SCIENCE
Open Access

Supporting Information

for *Adv. Sci.*, DOI 10.1002/advs.202302974

Highly Strain-Stable Intrinsically Stretchable Olfactory Sensors for Imperceptible Health Monitoring

Guodong Zhao, Jing Sun, Mingxin Zhang, Shanlei Guo, Xue Wang, Juntong Li, Yanhong Tong, Xiaoli Zhao, Qingxin Tang* and Yichun Liu*

Supporting information

Highly strain-stable intrinsically stretchable olfactory sensors for imperceptible health monitoring

Guodong Zhao, Jing Sun, Mingxin Zhang, Shanlei Guo, Xue wang, Juntong Li, Yanhong Tong, Xiaoli Zhao, Qingxin Tang**, and Yichun Liu*

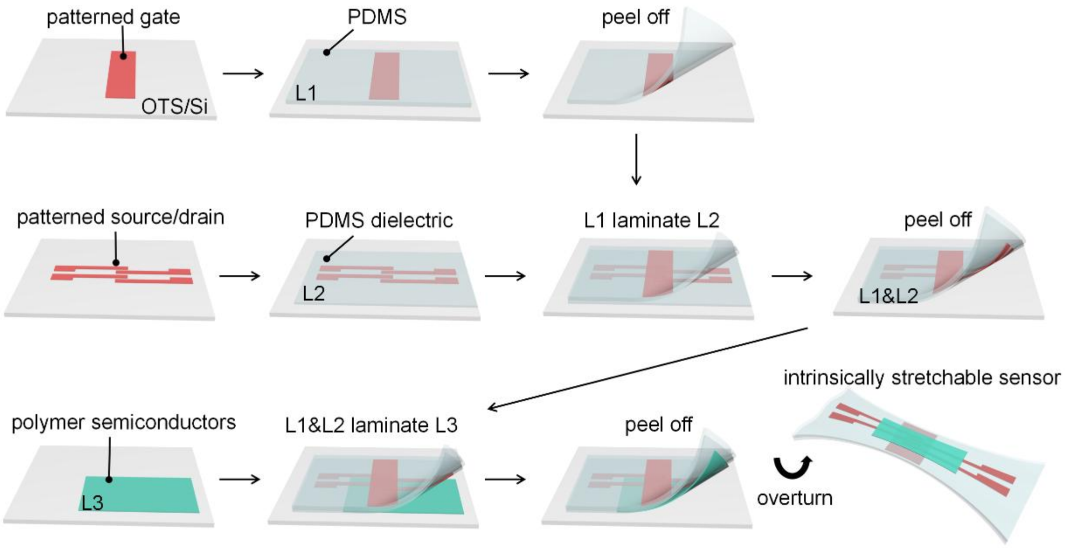

Figure S1. The detailed fabrication process of the intrinsically stretchable gas sensor.

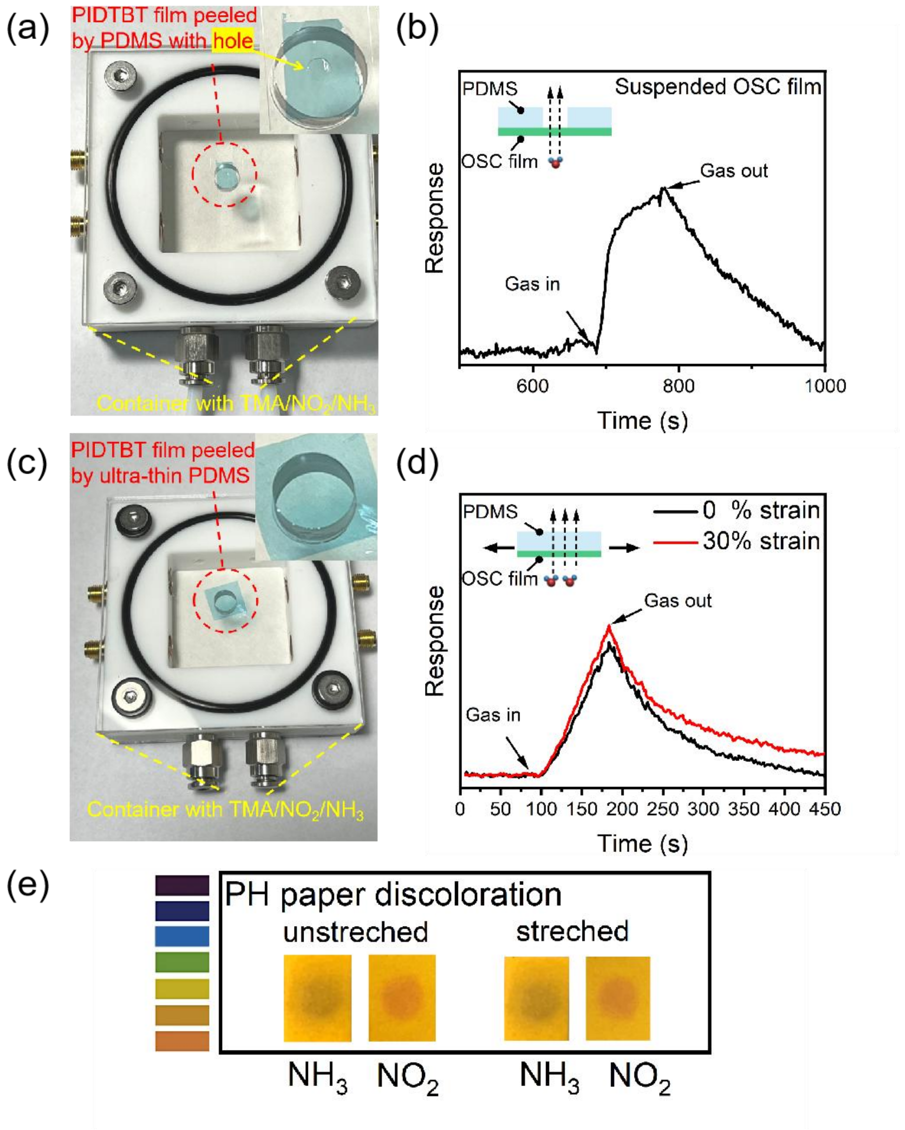

Figure S2. The real photograph of the container sealed by the two types of polymer semiconductor film (a, c) and the corresponding response curve to leakage of gas (b, d).

The gas permeability of the semiconductor is confirmed by sealing the container containing the gases (including TMA, NO_2, NH_3) with a polymer semiconductor film, and then checking the degree of gas leakage. The photograph of the container sealed by the polymer semiconductor is shown in Fig. S2a, c.

Herein, a stable atmospheric pressure gas environment can be obtained by slowly injecting the target gas into the container and then closing the two outlets. In order to avoid the influence of the disturbance of ambient gas on the leakage gas, we put the

semiconductor film-sealed container into a larger airtight tank (7000 ml). Then a low-flow vacuum pump (~500 ml/min, calibration by comparison with mass flow meter bubble velocity) is used to pump the gas from the tank into another gas detection chamber to detect changes in concentration. Due to the low flow rate, gas replenished from the outside of the tank for a short period (100s) has less effect on its internal gas concentration.

Two types of semiconductor thin films were used in the specific experiments. First, the suspended semiconductor films were used to confirm the gas permeability of the unstretched film, which can be obtained via the peel-off process using a PDMS film with small holes. As shown in Figure S2b, when the gas was injected into the semiconductor-sealed container, the sensor quickly exhibited a strong response and a slow recovery when the container was removed. This directly demonstrates the excellent gas permeability of unstretched polymer semiconducting films. However, the further stretched suspended films require PDMS films (self-supporting elastomeric films) with regular and small pores and smoother pore/film interfaces. It is currently still challenging to obtain high-quality through-holes in elastomer with a thickness of tens of micrometers, which is not the focus of this work.

The air permeability of stretched semiconductor films was determined using semiconductor films that were attached to ultra-thin and complete PDMS substrates. Because PDMS as an amorphous polymer has exhibited gas permeability in numerous previous works. As shown in Figure S2d, a significant response was also observed in the black line (unstretched film), but the time point of the response lagged behind the time point of gas injection. This may be because the thickness of PDMS reaches tens of microns, and gas molecules need to undergo slow diffusion or dissolution to diffuse out. When the film was stretched at 30% strain, the sensor's response increased slightly by ~10% (red line), compared to the former. This may be because the smaller thickness caused by stretching shortens the path of gas permeating the film. In summary, polymer semiconductor films are gas-permeable regardless of whether they are stretched or not, that is, gas molecules are allowed to enter the interior of the film through free diffusion and then pass through the film.

Further, the gas permeability of the polymer film to NH_3 and NO_2 was visually demonstrated using PH test paper. Specifically, a container containing a solution of concentrated nitric acid and ammonia was sealed with a polymer film, and PH paper was placed on top of the film. As NH_3 and NO_2 permeate the film, it can be observed that the pH test paper undergoes an obvious discoloration reaction corresponding to the acidity and alkalinity, as shown in Figure S2e. The color change of the pH test paper placed on the stretched and unstretched film was consistent, which may be related to the lower resolution. Nevertheless, these results still provide intuitive evidence that gases can permeate polymer semiconductor thin films.

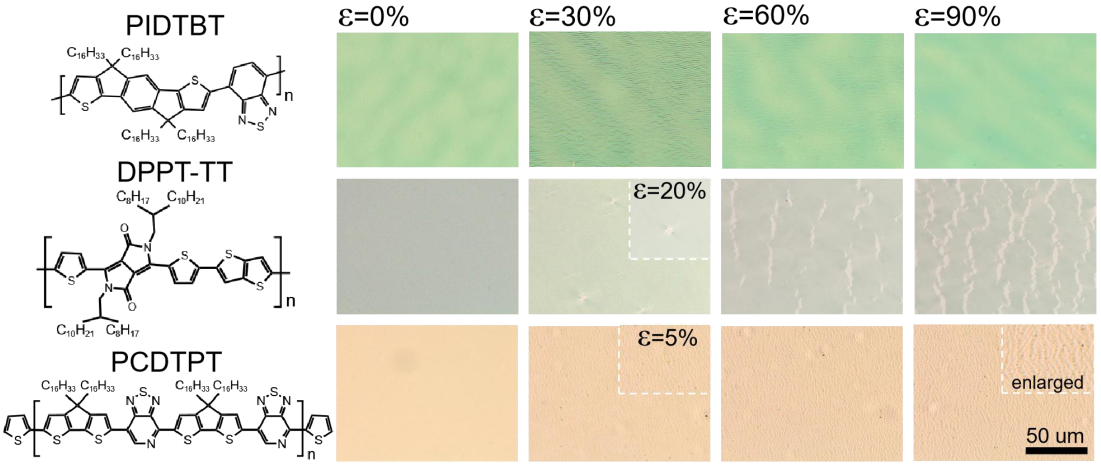

Figure S3 the Optical micrographs of polymer semiconductors in different stretched states. Insets show crack-onset strains of DPPT-TT and PCDTPT, respectively.

Figure S3 shows that the morphology of PIDBTT film is uniform without any cracks, even after stretching to 90%. DPPT-TT starts to crack at ~20% strain. As the strain increases, cracks with larger gaps appear. PCDTPT starts to crack at ~5% strain. As the strain increases, dense but small gap cracks appear.

Table S1 Comparison of this work with the reported stretchable gas sensor in terms of selectivity, operating temperature, stretchability, the limit of detection, and strain interference.

Stretchable method	Materials	Maximum strain	*Strain interference	Selectivity	*LOD (ppm)	Temp.	Ref.
Wave-structure	PbS QDs	90%	0.6	NO_2	1	RT	1
	PANI/MoS_2	30%	0.78	NH_3	0.5	RT	2
Porous network	P3HT&SEBS	40%	0.375	NO_2	2.45 ppb	RT	3
E-textile	rGO/ZnO NRs /Pd NPs/Ag NWs	100%	0.50	NO_2	5	RT	4
	rGo-ZnO	65%	0.45	NO_2	0.5	RT	5
Island-bridge structure	rGO/MoS_2	20%	1	NO_2	1	60	6
	MoS_2	70%	0	NO_2	5	RT	7
	rGO/MoS_2	40%	1.6	NO_2	200	RT	8
Slip structure	ZnO nanosheet	5%	10	NO_2	10	RT	9
Intrinsically stretchable	hydrogel	100%	1.4	NO_2	0.5	RT	10
	organohydrogel	>100%	0.6	NO_2	1	RT	11
	hydrogel	>100%	0.07-	NO_2	0.08	RT	12
	inorganic nanofiber	50%	38	NO_2	0.02	RT	13
	([EMIM]+[TFSI]−) -TPU	100%	0.008	toluene	1	RT	14
	([EMIM]+[TFSI]−) -TPU	50%	0.36	NO_2	0.25	RT	15
	rGO/AgNWs-PU	60%	0.33	NO_2	5	RT	16
	rGO/PDDA	30%	0.67	NO_2	1	RT	17
	**Polymer semiconductor**	**90%**	**0.055**	**TMA**	**0.3 ppb**	**RT**	**This work**

Note: "Strain interference" is defined as the ratio of the change in response due to tensile strain to the change in strain and can be expressed as $\Delta R/\Delta\varepsilon$. "LOD" extracts from the calculated/ theoretical LOD from the literature, or extracts from the lowest concentration actually detected when there is no calculated LOD.

Reference for Table S1

[1] Song, Z.; Huang, Z.; Liu, J.; Hu, Z.; Zhang, J.; Zhang, G.; Yi, F.; Jiang, S.; Lian, J.; Yan, J.; et al. Fully Stretchable and Humidity-Resistant Quantum Dot Gas Sensors. *ACS Sensors* **2018**, *3* (5), 1048-1055.

[2] Yan, H.; Zhong, M.; Lv, Z.; Wan, P. Stretchable Electronic Sensors of Nanocomposite Network Films for Ultrasensitive Chemical Vapor Sensing. *Small* **2017**, *13*, 1701697.

[3] Liu, C.; Wu, M.; Gao, L.; Liu, H.; Yu, J. Nanoporous polymer films based on breath figure method for stretchable chemiresistive NO_2 gas sensors. *Sensors and Actuators B: Chemical* **2022**, *371*, 132540.

[4] Thanh Hoang Phuong, D.; Qui Thanh Hoai, T.; Adem, S.; Nguyen Thuy, H.; Woochul, Y.; Jin-Seo, N. Highly Deformable Fabric Gas Sensors Integrating Multidimensional Functional Nanostructures. *ACS Sensors* **2020**, 5, 2255.

[5] Li, W.; Chen, R.; Qi, W.; Cai, L.; Sun, Y.; Sun, M.; Li, C.; Yang, X.; Xiang, L.; Xie, D.; et al. Reduced Graphene Oxide/Mesoporous ZnO NSs Hybrid Fibers for Flexible, Stretchable, Twisted, and Wearable NO_2 E-Textile Gas Sensor. *ACS Sensors* **2019**, *4*, 2809-2818.

[6] Li, Y.; Ning, Y.; Jia, Z.; Zheng, C.; Xinyang, Y.; Xueyi, Z.; Hongli, Z.; Huanyu, C. Novel gas sensing platform based on a stretchable laser-induced graphene pattern with self-heating capabilities. *Journal of Materials Chemistry A* **2020**, 8, 6487.

[7] Md Ashraful, I.; Hao, L.; Seokjin, M.; Sang Sub, H.; Hee-Suk, C.; Jinwoo, M.; Changhyeon, Y.; Tae-Jun, K.; Kyu Hwan, O.; YounJoon, J.; et al. Vertically Aligned 2D MoS_2 Layers with Strain-Engineered Serpentine Patterns for High-Performance Stretchable Gas Sensors: Experimental and Theoretical Demonstration. *ACS Applied Materials & Interfaces* **2020**,12, 53174.

[8] Ning, Y.; Zheng, C.; Han, L.; Li, Y.; Jia, Z.; Xiaoqi, Z.; Yong, C.; Zhendong, L.; Hongli, Z.; Huanyu, C. Stretchable, ultrasensitive, and low-temperature NO_2 sensors based on MoS_2@rGO nanocomposites. *Materials Today Physics* **2020**, 100265.

[9] Park, J.; Kim, J.; Kim, K.; Kim, S. Y.; Cheong, W. H.; Park, K.; Song, J. H.; Namgoong, G.;

Kim, J. J.; Heo, J.; et al. Wearable, wireless gas sensors using highly stretchable and transparent structures of nanowires and graphene. *Nanoscale* **2016**, *8*, 10591-10597.

[10]Zixuan, W.; Xing, Y.; Jin, W. Conductive Hydrogel- and Organohydrogel-Based Stretchable Sensors. *ACS Applied Materials & Interfaces* **2021**, 13, 2128.

[11] Wu, Z.; Rong, L.; Yang, J.; Wei, Y.; Tao, K.; Zhou, Y.; Yang, B. R.; Xie, X.; Wu, J. Ion-Conductive Hydrogel-Based Stretchable, Self-Healing, and Transparent NO_2 Sensor with High Sensitivity and Selectivity at Room Temperature. *Small* **2021**, *17*, e2104997.

[12] Wu, J.; Wu, Z.; Huang, W.; Yang, X.; Liang, Y.; Tao, K.; Yang, B. R.; Shi, W.; Xie, X. Stretchable, Stable, and Room-Temperature Gas Sensors Based on Self-Healing and Transparent Organohydrogels. *ACS Appl Mater Interfaces* **2020**, *12*, 52070-52081.

[13] Wang, B.; Thukral, A.; Xie, Z.; Liu, L.; Zhang, X.; Huang, W.; Yu, X.; Yu, C.; Marks, T. J.; Facchetti, A. Flexible and stretchable metal oxide nanofiber networks for multimodal and monolithically integrated wearable electronics. *Nat Commun* **2020**, *11*, 2405.

[14] Jin, M. L.; Park, S.; Kim, J. S.; Kwon, S. H.; Zhang, S.; Yoo, M. S.; Jang, S.; Koh, H. J.; Cho, S. Y.; Kim, S. Y.; et al. An Ultrastable Ionic Chemiresistor Skin with an Intrinsically Stretchable Polymer Electrolyte. *Adv Mater* **2018**, *30*, e1706851.

[15] Ming Liang, J.; Sangsik, P.; Hyukmin, K.; Hyeong-Jun, K.; Min, G.; Chao, T.; Soo-Yeon, C.; Yunpyo, K.; Shuye, Z.; Xinlin, L.; et al. Scalable Superior Chemical Sensing Performance of Stretchable Ionotronic Skin via a π-Hole Receptor Effect. Advanced Materials 2021, 33, 2007605.

[16] Luan, Y.; Zhang, S.; Nguyen, T. H.; Yang, W.; Noh, J.-S. Polyurethane sponges decorated with reduced graphene oxide and silver nanowires for highly stretchable gas sensors. *Sensors and Actuators B: Chemical* **2018**, *265*, 609-616.

[17] Adeela, H.; Atanu, B.; Arsalan, Z.; Dong-Bin, M.; Surjeet, K.; Sajal, S.; Nae-Eung, L. A Skin-Inspired Substrate with Spaghetti-Like Multi-Nanofiber Network of Stiff and Elastic Components for Stretchable Electronics. *Advanced Functional Materials* **2020**, *30*, 2003540.
